# Metropolitan Hospital Sunday Fund

**Published:** 1898-08-13

**Authors:** 


### METROPOLITAN HOSPITAL SUNDAY FUND.

ANNUAL MEETING.

Sir Sydney Waterlow presided at the Mansion House, on Wednesday, 3rd inst., in the absence of the Lord Mayor, at the meeting of the Council of the Metropolitan Hospital Sunday Fund, to receive the report of the Committee of Distribution for the coming year. Awards are made this year to 186 institutions—132 hospitals and 34 dispensaries—the total amount available for distribution, after allowing for liabilities and current expenses, being £35,000. Of this, £34,304 is recommended to be paid. One hundred and ninety-five institutions made applications for grants, and the attendance was invited of deputations from the governing bodies of 17 of these. In six cases, of which two applications were withdrawn, no recommendation was made. Deputations from 12 hospitals and one dispensary conferred with the committee, and in most cases explanations were given modifying the causes of criticism, and enabling the bases of awards to be raised. The committee note that arrangements for restricting the benefits of the out-patient system to those less able to afford to pay for medical treatment have been made at most of the large hospitals and at many of the smaller institutions, and that the practice of making a small charge to out-patients is increasing. As usual, 5 per cent. of the sum collected—amounting this year to about £2,000—has been set aside for the purchase of surgical appliances.

In proposing the adoption of the report, and the payment of the several awards, the Chairman congratulated the Council on the amount collected—£38,741 in all—which, although less than in previous years must, he thought, be considered satisfactory, taking into account the various difficulties against which they had had to contend. Many large contributions had been transferred to the Prince of Wales's Fund, or divided with it, but he felt assured that appeals through ministers of religion would continue to have force and influence. It certainly was a religious duty to afford succour and relief to the poor and sick and suffering; and it was a cause of thankfulness that the various churches and chapels succeeded in collecting so large a sum on Hospital Sunday. Each year added to the number of institutions among whom their Fund had to be distributed, and each year more time and, if possible, more trouble was devoted to examining and analysing the accounts before arriving at the decision which they recommended for adoption. The conferences they had had with the deputations from various hospitals had undoubtedly been beneficial, and as a result of the committee's wide experience many of these had been persuaded to improve their arrangements in certain particulars. One of the subjects of discussion had been the possible abuse of the out-patient system, and while the committee would continue to recommend the appointment of an experienced official with a view to the supervision of the out-patients in this respect, yet he was convinced that in but a very small percentage of cases did abuse arise, the recipients of relief belonging, in the vast majority of cases, to the classes it was intended to benefit.

Dr. J. G. Glover, who seconded the adoption of the report, was, nevertheless, inclined to differ somewhat from its tone with regard to the falling off of £5,000 or more in their distribution. Personally, while admiring the confidence and hopefulness displayed, he was conscious of some dissatisfaction and discouragement, since it was necessary to go back eleven or twelve years—in fact, to 1885—to find so small a total. He, therefore, should be inclined to recommend the committee to enter into a minute examination of the causes which had contributed to the falling off. He thought it would not do to be too ready to attribute it altogether to the existence of another fund—and he was sure the Prince of Wales would be the first to regret it if his fund should cause any shortage in theirs—but they should try and find some other explanation.

The Hon. Sydney Holland, as one of those responsible for the management of the hospitals, said he looked to that and similar Funds to relieve those who gave their time and attention to the work of administration from the disagreeable duty of writing begging letters. He felt most strongly that the public ought to support them much more largely than they did. So many seemed content to consider that it was other people's duty to contribute. He was inclined to look to some such idea as the organisation, by the ministers of religion or otherwise, of some form of a voluntary rate, to be distributed by that Council. Their needs were constantly becoming greater; the number of hospitals was increasing; they did not care now to work their nurses as they used to do; and they wanted better medical appliances so as to get the best possible results. Undoubtedly, and he wished to emphasise and urge this upon them, unless the hospitals were much more largely supported than at present, there were evil times before them. In conclusion, there was a point of detail he wished to bring before the committee, and that was the question of the inclusion of interest on mortgages under the head of administration, thus making the percentage of some hospitals much less favourable in appearance than that of others really no better managed. He thought—and the chairman agreed with him and promised that the matter should be discussed at their next meeting—that such interest should go under the head of maintenance in the same way as rent.

Lord Stamford moved a resolution, which was seconded by Canon Fleming, thanking Sir Sydney Waterlow and the Committee of Distribution for the discretion exercised in interviewing deputations and the care bestowed on the preparation of the awards recommended. He referred to the question of the possible abuse of the out-patient system, and hoped the duties of the inquiry officers would be not only to detect those who were not entitled to seek relief in that way, but that specially skilled officials would be selected with a view to helpfulness in all cases, to indicate to patients the proper quarters to apply to, and the most appropriate hospital, dispensary, convalescent home, and so forth, for their case. He felt that thus they would be beginning a really important movement.

Sir Edmund Currie moved that the thanks of the Council be given to the editors of newspapers who had pleaded the cause of the hospitals and advocated the cause of the Fund.

Sir Henry Burdett seconded this most cordially. It was not a perfunctory resolution, they meant it; but he was convinced that if the Fund was to be maintained in the neighbourhood of £40,000 a year steps must be taken to direct the attention of the various editors of the Hospital Sunday Fund, and to facts and statistics relating to it. He had been much struck that year by the silence of the newspapers on the subject contrary to their custom, and by the consequent ignorance of the public regarding the date of the collection. This was not due to want of willingness on the part of the editors but to want of material, and the Council should make it their business to supply them with information, so that they might help them by publicity in their columns. He knew that many clergymen were not desirous of too much publicity, but between too much and not enough there was a great gulf. He was justified in his opinion as to the need for help from the Press by the figures when efforts had been made to enlist it. As an example of the inestimable services that could be rendered by the Press he would point to the case of the London Hospital, where they had taken the matter into their own hands, and not only by publicity but by personal service they had rendered the very greatest help. That he considered a sign of great hopefulness for the future, and felt sure that voluntary support forthcoming for the hospitals would be adequate. He confessed to feeling some dread of a State-supported system, as apt to be productive of more inefficiency, less care for the sick, and more abuses.

After a vote of thanks to the Lord Mayor as president and treasurer of the Fund,

Dr. Glover brought forward a motion requesting the Distributing Committee to inquire into and consider the causes of the diminution of the collections for the fund for 1898, and report to the Council at its next meeting. He thought they ought to get at the bottom of the matter, and that the committee was in a position to find out the facts, and so express a well-grounded opinion.

Mr. Thomas Christy said his belief was that the falling off was due to the managers of the Prince of Wales's Fund, who had laid their plans with such consummate ability as to capture many regular subscribers, and arrange that their money should come in from year to year.

To this Sir Henry Burdett replied that he had gone into the accounts and had compared them with previous years, and he could state positively that, with the exception of four, where the circumstances were special and the reasons apparent, every hospital in London had been more prosperous than in previous years. Whatever the cause of the falling off in the Hospital Sunday Fund this year, he was quite sure it was not owing to the Prince of Wales's Fund. In reality they were touching but the borders of the field open to them from which to obtain support; there still remained to be tapped the immense mass of indifferent people who, as ever, were content to get the benefits provided and give nothing in return. One of the causes, in his opinion, of the want of increase in the revenue of charities was the operation of the Limited Liability Acts, which, by withdrawing the personal element from business firms, had done mischief to many matters dear to the City and important to the welfare of the whole community. When a firm became a limited liability company—and there were now more than 10,000 in existence—charity suffered; with the disappearance of the men who had guided these firms disappeared a fruitful source of income for the hospitals. It had been suggested that a voluntary rate might be levied, but very many of these companies were compound householders and could not be reached because they were not on the rate books, the block of buildings in which a large number of companies had their offices being often owned by one person. It was difficult to appeal to a limited company; it had no soul, nor body, nor heart, and though the directors were men, and had often actually power under their articles of association to make contributions for charitable purposes, they sheltered themselves under their corporate capacity.

The Chairman, in reply, hoped that the burden of such an investigation as that suggested would not be thrown on the committee. He was very glad so much had been collected by the Prince's Fund; the money had been judiciously applied. He was inclined to disagree with the suggestion that limited companies should be asked to make contributions. After voting away other people's money shareholders were apt to comfort themselves with the idea that they had done their duty, but his notion had always been that charity was a personal obligation.

The resolution was put to the meeting and lost.

### PARTICULARS OF AWARDS.

The following is the report of the Committee of Distribution adopted by the Council on the 3rd inst:—

"The Committee of Distribution recommend awards this year to the 186 institutions shown in the appendix attached hereto, being an increase of 81 since the first awards were made in 1873, and two less than last year. The total amount available for distribution, after allowing for liabilities and the usual current expenses, is £35,000. Of this total £34,304 is now recommended to be paid to 132 hospitals and 54 dispensaries.

"This year 195 institutions have made applications for grants from the funds at your disposal. Of this number it was found necessary to invite the attendance of deputations from the governing bodies of 17. In six of these cases, including two applications which were withdrawn, your committee are not able to make any recommendation. Deputations from 12 hospitals and from one dispensary conferred with your committee, and in nearly every case your committee received explanations modifying the causes of adverse criticism, and enabling them to raise the bases of awards as now recommended.

"Your committee are glad to be able to state that arrangements for restricting the advantages of the out-patient system to the poor have been made at nearly all the large and at some of the smaller hospitals. The practice of making a small charge to out-patients is rather on the increase. They are of opinion that the conferences which have taken place have been very advantageous to all parties attending them.

"Five per cent. of the total sum collected—viz., about £2,000, is set apart to purchase surgical appliances (in obedience to Law XII.), in monthly proportions during the ensuing year.

"In compliance with an order of the Council, and for the special use of its members, tables of statistics have been prepared as usual, showing an analysis of the number of beds in hospitals, the cost of patients both at hospitals and dispensaries, the proportionate expense of management, as well as other valuable information, and copies are sent to every member of the Council.

"The amount available for distribution is less than that distributed last year, but your committee feel that, following on a Jubilee year, in which strong appeals were made in other directions, the collections this year on Hospital Sunday maintain a fair average, and that the confidence of the public in the Metropolitan Hospital Sunday Fund is well sustained.

"Your committee recommend that all payments to the Fund after this date be carried to the credit of next year's Fund."

### Twenty-eight General Hospitals.

Charing Cross Hospital, £9[illegible]0; French Hospital, £262 10s.; German Hospital, £457 10s.; Great Northern Central Hospital, £412 10s.; Guy's Hospital, £1,125; Hampstead Hospital, £120; Italian Hospital, £47 5s.; King's College Hospital, £1,200; London Hospital, £3,525; London Homœopathic Hospital, £375; Phillips Memorial Homœopathic Hospital, £26 5s.; London Temperance Hospital, £577 10s.; Metropolitan Hospital, £487 10s.; Mildmay Mission Hospital, £225; Miller Hospital and Royal Kent Dispensary, £202 10s.; North-West London Hospital, £300; Poplar Hospital, £450; Royal Free Hospital, £750; St. George's Hospital, £975; SS. John and Elizabeth Hospital, £60; St. Mary's Hospital, £1,[illegible]87; Seamen's Hospital Society, £787 10s.; The Middlesex Hospital, £1,575; University College Hospital, £1,012 10s.; Walthamstow, £60; West Ham Hospital, £270; West London Hospital, £412 10s.; Westminster Hospital, £975.

### Special Hospitals.

Five Chest Hospitals.—City of London Hospital for Diseases of the Chest, Victoria Park, £731 5s.; Hospital for Consumption, Brompton, £1,350; North London Consumption Hospital, Hampstead, £225; Royal Hospital for Diseases of the Chest, City Road, £225; Royal National Hospital for Consumption (Ventnor), £187 10s.

Sixteen Children's Hospitals.—Alexandra Hospital for Hip Disease, W.C., £123 15s.; Barnet Home Hospital, £33 15s.; Belgrave Hospital for Children, S.W., £105; Cheyne Hospital for Incurable Children, S.W., £97 10s.; East London Hospital for Children, Shadwell, E., £375; Evelina Hospital for Sick Children, Southwark, S.E., £337 10s.; Home for Incurable Children, Maida Vale, W., £52 10s.; Home for Sick Children, Sydenham, S.E., £108 15s.; Hospital for Sick Children, Great Ormond Street, W.C., £525; North-Eastern Hospital for Children, Hackney Road, N.E., £225; Paddington Green Hospital for Children, W., £150; St. Mary's, Plaistow, E., £93 15s.; St. Monica's, Brondesbury, N.W., £75; Victoria Hospital for Children, King's Road, Chelsea, S.W., £412 10s.; Victoria Home, Margate, £22 10s.; "The Vine," Sevenoaks, £30.

Five Lying-in Hospitals.—British Lying-in Hospital, Endell Street, W.C., £18 15s.; Clapham Maternity Hospital, £22 10s.; East-End Mothers' Home, £60; General Lying-in Hospital, Lambeth, S.E., £37 10s.; Queen Charlotte's Lying-in Hospital, Marylebone Road, W., £150.

Six Hospitals for Women.—Chelsea Hospital for Women, S.W., £281 5s.; Hospital for Women, Soho Square, W., £2[illegible]2 10s.; Grosvenor Hospital for Women and Children, Vincent Square, S.W., £37 10[illegible].; New Hospital for Women, Euston Road, W.C., £172 10s.; Royal Hospital for Children and Women, Lambeth, S.E., £168 15s.; Samaritan Free Hospital, Lower Seymour Street, W., £375.

### Twenty-five Other Special Hospitals.

Cancer Hospital, Brompton, S.W., £450; London Fever Hospital Islington, N., £750; Gordon Hospital for Fistula, Vauxhall Bridge Road S.W., £27 15s.; St. Mark's Hospital for Fistula, City Road, E.C., £67 10s.; National Hospital for Diseases of the Heart and Paralysis, Soho Square, W., £52 10s.; Female Lock Hospital, Harrow Road, W., £13[illegible] 15s.; Hospital for Epilepsy, Paralysis and other Diseases of the Nervous System, Regent's Park, N.W., £48 15s.; National Hospital for the Paralysed and Epileptic, W.C., £645; West-end Hospital for Diseases of the Nervous System, £82 10s.; Central London Ophthalmic Hospital, Gray's Inn Road, W.C., £15; Royal Eye Hospital, St. George's Circus, S.E., £90; Royal London Ophthalmic Hospital, Moorfields, E.C., £412 10s.; Royal Westminster Ophthalmic Hospital, Charing Cross, W.C., £75; Western Ophthalmic Hospital, Marylebone Road, W., £33 15s.; City Orthopædic Hospital, Hatton Garden, E.C., £41 5s.; National Orthopædic Hospital, Great Portland Street, W., £67 10s.; Royal Orthopædic Hospital, Oxford Street, W., £52 10s.; Royal Sea-Bathing Hospital, Margate, £225; St. Peter's Hospital for Stone, Covent Garden, £30; Central London Throat and Ear Hospital, Gray's Inn Road, W.C., £30; London Throat Hospital, Great Portland Street, W., £22 10s.; Metropolitan Ear and Nose Hospital, nil; Royal Ear Hospital, Frith Street, W., £7 10s.; Dental Hospital for London, Leicester Square, W.C., £93 15s.; National Dental Hospital, 149, Great Portland Street, W., £86 5s.

### Twenty-eight Convalescent Hospitals.

Metropolitan Convalescent Institution, Sackville Street, W., £450; Bexhill Convalescent Home, Sackville Street, W., £225; All Saints' Convalescent Hospital, Eastbourne, £412 10s.; Mrs. Gladstone's Convalescent Home, Woodford, £60; Hahnemann Convalescent Home, Bournemouth, £26 5s.; Hanwell Convalescent Home, £17 5s.; Herbert Convalescent Home, Bournemouth, £15; Herne Bay Baldwin-Brown Convalescent Home, £22 10s.; Herne Bay Mothers' Home, £33 15s.; Homœopathic Convalescent Home, Eastbourne, £15; King's College Hospital Convalescent Home, Hemel Hempstead, £48 15s.; Mrs. Kitto's Convalescent Home, Reigate, £60; London and Brighton Female Convalescent Home, £15; Mary Wardell Convalescent Home for Scarlet Fever, £75; Morley House Convalescent Home for Working Men, £67 10s.; St. Andrew's Convalescent Hospital, Clewer, £112 10s.; St. Andrew's Convalescent Home, Folkestone, £202 10s.; St. John's Home for Convalescent and Crippled Children, Brighton, £60; St. Joseph's Convalescent Home, Bournemouth, £37 10s.; St. Leonards-on-Sea Convalescent Home for Poor Children, £67 10s.; St. Michael's Convalescent Home, Westgate-on-Sea, £18 15s.; Seaside Convalescent Hospital, Seaford, £75; Warley Convalescent Cottage Hospital, Essex, £5 5s.; Ascot Priory Convalescent Home, £45; Police Seaside Home, Brighton, £30; St. Mary's Convalescent Home, Shortlands, £11 5s.; Chelsea Hospital for Women Convalescent Home, St. Leonards, £30; Deptford Medical Mission Convalescent Home, Bexhill, £15.

### Thirteen Cottage Hospitals.

Beckenham Cottage Hospital, £37 10s.; Blackheath and Charlton Cottage Hospital, £52 10s.; Bromley, Kent, Cottage Hospital, £63 15s.; Chislehurst, Sidcup, and Cray Valley Cottage Hospital, £30; Eltham Cottage Hospital, £24 15s.; Enfield Cottage Hospital, £33 15s.; Epsom and Ewell Cottage Hospital, £45; Hounslow Cottage Hospital, £13 10s.; Mildmay Cottage Hospital, £52 10s.; Reigate and Redhill Cottage Hospital, £45; Sidcup Cottage Hospital, £27 15s.; Wimbledon Cottage Hospital, £26 5s.; Woolwich and Plumstead Cottage Hospital, £26 5s.

### Seven Institutions.

Establishment for Gentlewomen, Harley Street, £93 15s.; St. Saviour's Hospital and Nursing Home, £86 5s.; National Sanatorium for Consumption, Bournemouth, £45; Invalid Asylum, Stoke Newington, £45; Firs Home, Bournemouth, £15; St. Catherine's Home, Ventnor, £22 10s.; Friedenheim Home for the Dying, £187 10s.

### Fifty-five Dispensaries.

Battersea Provident Dispensary, £60; Bloomsbury Provident Dispensary, £7 10s.; Brixton, &c., Dispensary, £45; Brompton Provident Dispensary, £18; Camberwell Provident Dispensary, £75; Camden Provident Dispensary, £9; Chelsea, Brompton, and Belgrave Dispensary, £36; Chelsea Provident Dispensary, £11 5s.; Child's Hill Provident Dispensary, £7 10s.; City Dispensary, £75; City of London and East London Dispensary, £18; Clapham General and Provident Dispensary, £19 10s.; Deptford Medical Mission, £15; Eastern Dispensary, £22 10s.; East Dulwich Provident Dispensary, £11 5s.; Farringdon General Dispensary, £30; Finsbury Dispensary, £24; Forest Hill Dispensary, £22 10s.; Hackney Provident Dispensary, £6; Hampstead Provident Dispensary, £33 15s.; Holloway and North Islington Dispensary, £45; Islington Dispensary, £33 15s.; Kensal Town Provident Dispensary, £3 15s.; Kensington Dispensary, £48 15s.; Kilburn, Maida Vale, and St. John's Wood Dispensary, £24; Kilburn Provident Medical Institution, £26 5s.; London Dispensary, £15; London Medical Mission, Endell Street, £52 10s.; Margaret Street Infirmary for Consumption, nil; Metropolitan Dispensary, £37 10s.; Notting Hill Dispensary and Maternity (Provident), £7 10s.; Paddington Provident Dispensary, £24 15s.; Pimlico Provident Dispensary (Lupus Street), £11 5s.; Portland Town Dispensary, £12; Public Dispensary, £33 15s.; Queen Adelaide's Dispensary, £16 10s.; Royal General Dispensary, £30; Royal Pimlico Provident Dispensary, £41 5s.; Royal South London Dispensary, £48 15s.; St. George's and St. James's Dispensary, £33 15s.; St. George's, Hanover Square, Provident Dispensary, £26 5s.; St. John's Wood and Portland Town Provident Dispensary, £27 15s.; St. Marylebone General Dispensary, £37 10s.; St. Pancras and Northern Dispensary, £30; South Lambeth, Stockwell, and North Brixton Dispensary, £33 15s.; Stamford Hill, Stoke Newington, &c., Dispensary, £33 15s.; Tower Hamlets Dispensary, £27; Walworth Provident Dispensary, £8 5s.; Wandsworth Common Provident Dispensary, £6 15s.; Westbourne Provident Dispensary and Maternity, £7 10s.; Western Dispensary, £[illegible]0; Western General Dispensary, £97 10s.; Westminster General Dispensary, £19 10s.; Whitechapel Provident Dispensary, £15; Woolwich Provident Dispensary, £7 10s.

